# Effect of Pioglitazone on the Fructose-Induced Abdominal Adipose Tissue Dysfunction

**DOI:** 10.1155/2012/259093

**Published:** 2012-10-02

**Authors:** Ana Alzamendi, Andrés Giovambattista, María E. García, Oscar R. Rebolledo, Juan J. Gagliardino, Eduardo Spinedi

**Affiliations:** ^1^Neuroendocrine Unit IMBICE, CICPBA, and CONICET, P.O. Box 403, 1900 La Plata, Argentina; ^2^CENEXA, UNLP and CONICET and PAHO/WHO Collaborating Centre, 1900 La Plata, Argentina

## Abstract

*Aim*. To test the potential role of PPAR**γ** in the endocrine abdominal tissue dysfunction induced by feeding normal rats with a fructose rich diet (FRD) during three weeks. *Methodology*. Adult normal male rats received a standard commercial diet (CD) or FRD, (10% in drinking water) without or with pioglitazone (PIO) (i.p. 0.25 mg/Kg BW/day; CD-PIO and FRD-PIO). Thereafter, we measured circulating metabolic, endocrine, and oxidative stress (OS) markers, abdominal adipose tissue (AAT) mass, leptin (LEP) and plasminogen activator inhibitor-1 (PAI-1) tissue content/expression, and leptin release by isolated adipocytes incubated with different concentrations of insulin. *Results*. Plasma glucose, insulin, triglyceride, TBARS, LEP, and PAI-1 levels were higher in FRD rats; PIO coadministration fully prevented all these increments. AAT adipocytes from FRD rats were larger, secreted a higher amount of LEP, and displayed decreased sensitivity to insulin stimulation; these effects were significantly ameliorated by PIO. Whereas AAT LEP and PAI-1 (mRNA) concentrations increased significantly in FRD rats, those of insulin-receptor-substrate- (IRS-) 1 and IRS-2 were reduced. PIO coadministration prevented FRD effects on LEP, PAI-1, and IRS-2 (fully) and IRS-1 (partially) mRNAs in AAT. *Conclusion*. PPAR**γ** would play a relevant role in the development of the FRD-induced metabolic-endocrine dysfunction.

## 1. Introduction

The annual *per capita* consumption of fructose has drastically risen in the USA in recent decades [1], and some authors consider that this increased consumption could actively contribute to the development of the current epidemics of obesity, type 2 diabetes, and metabolic syndrome (MS) [2, 3]. Several studies have also demonstrated that administration of a fructose-rich diet (FRD) to normal rats induces the development of features characteristic of the human MS phenotype [4–7]. For these reasons, US Dietary Guidelines recommend limiting calorie intake (which includes both added sugar and solid fat) to 13% of energy requirement [8]. 

Although the precise mechanism whereby FRD induces the features of MS is still controversial, it has been suggested that an increased rate of oxidative stress (OS) is actively involved [9–13]. In this regard, we have previously reported increased OS in abdominal adipose tissue (AAT) [14] and impaired adipoinsular axis function [14, 15] in normal rats fed an FRD for 3 weeks. 

Despite that the metabolism of fructose by pancreatic *β* cells is poor or null [16, 17], fructose can potentiate insulin secretion induced by physiological concentrations of glucose [18]. Because fructose affects different cells of the digestive tract [19], liver [20], and adipose tissue [21, 22], it can indirectly modulate pancreatic *β*-cell function through the release of metabolites and hormones/adipokines by those tissues. The facts that the intake of an FRD increases serum triglyceride and insulin levels and impairs glucose tolerance supports this assumption [14, 15]. All these alterations could be consequences of the enhancement in the OS rate [14] and/or the impairment of tissue insulin sensitivity induced by fructose [6, 23–25]. 

On account that PPAR-*γ* plays an important role in the control of tissue-insulin sensitivity, we currently studied the involvement of those receptors in the FRD-induced AAT endocrine dysfunction. For this aim, we investigated the effect of simultaneous administration of FRD and pioglitazone (PIO), an effective PPAR-*γ* agonist [26, 27] on (a) circulating concentrations of metabolic, endocrine, and OS markers, (b) morphometric characteristics of AAT adipocytes, (c) *in vitro* leptin release by isolated AAT adipocytes, and (d) expression of adipokines and of intracellular insulin mediators (insulin-receptor-substrate- (IRS-) 1 and IRS-2) in the AAT.

## 2. Materials and Methods

### 2.1. Animals and Experimental Design

Normal adult male Wistar rats (180–200 g body weight) were kept in a temperature-controlled environment (23°C) on a fixed 12-hour light/dark cycle and fed *ad libitum* for one week (stabilization period) with a standard commercial diet (rat chow, Ganave, Argentina). Thereafter, rats were randomly divided into four groups (30 animals each) and fed *ad libitum* for 3 weeks with (i) commercial standard chow and tap water (control diet group; CD), (ii) CD diet plus 10% fructose (w/v; Carlo Erba Reagents, Italy) in drinking water (FRD group), (iii) CD diet plus daily injection (i.p.) of PIO (0.25 mg/Kg BW; Lab. Phoenix, Argentina) [28] (CD-PIO), and (iv) FRD diet plus PIO (FRD-PIO). Rats from both CD and FRD groups also received daily i.p. injections of 300 *μ*L of sterile vehicle only. This study protocol complies with international regulations concerning the ethical use and care of animals. 

### 2.2. Blood Measurements

Nonfasting animals were killed (between 08:30 and 09:00 h), trunk blood was collected into EDTA-coated tubes, rapidly centrifuged (4°C at 3,000 rpm), and plasma samples were immediately analysed or stored at −20°C. Commercial assay kits were used to measure plasma levels of glucose (Bio System Lab., Argentina), triglyceride (TG) (Wiener Lab., Argentina), and nonesterified fatty acids (NEFA; Randox Laboratories Ltd., UK). Plasma insulin concentration was determined by radioimmunoassay (RIA) [29] and lipid peroxidation by measuring thiobarbituric acid reactive substances (TBARS) [14]. Amount of TBARS formed was calculated by the extinction coefficient for the malonaldehyde-TBA complex of 1.56 × 10^5^ (mol/L)^−1^ cm^−1^ and expressed as pmol of TBARS per mg of plasma protein (measured with the Bio-Rad Protein Assay kit; Bio-Rad Lab, RC, USA). Leptin (LEP) concentration in plasma and in the incubation medium samples as well as in AAT extracts was measured by a validated specific RIA (standard curve 0.04–15 ng/mL) [30]. The coefficients of variation (CV) intra- and interassay of insulin and LEP RIAs were 3–7% and 5–9%, respectively. Since plasminogen activator inhibitor-1 (PAI-1) is another key marker of AAT dysfunction [15], its circulating levels were also measured using a commercial ELISA kit (American Diagnostica, Inc., CT, USA, IMUCLONE Cat. no. 601; standard curve 1–20 ng/mL, CVs intra- and interassay were 0.5–2% and 4–9%, resp.). 

### 2.3. Histological Studies

AAT pads were removed and immediately fixed in 4% paraformaldehyde (in 0.2 M phosphate buffer), at 4°C for a maximum of 3 days. Tissues were then washed with 0.01 M PBS, immersed in 70% ethanol, and thereafter embedded in paraffin. Sections of 4 *μ*m were taken from different levels of the blocks and stained with haematoxylin eosin. Quantitative morphometric analysis was performed using a Jenamed 2 Carl Zeiss light microscope, a RGB CCD Sony camera, and OPTIMAS software (Bioscan Incorporated, Edmons, WA, USA) (40x objective). For each AAT sample, 1 section and 3 levels were selected (*n* = 4/5 animals per group). Systematic random sampling was used to select 10 fields for each section and a minimum of 100 cells per group were counted. We then measured adipocyte diameter, whereas cell volume was calculated by the formula 4/3*π*·r^3^ [31].

### 2.4. Isolation and Incubation of Adipocytes from AAT

Adipocytes were isolated from preweighed AAT by a minor modification of the Rodbell procedure [29]. Briefly, fat pads were transferred into sterile plastic tubes containing Krebs-Ringer-MOPS medium with 1% BSA (Sigma Chem. CO, MO, USA) (w/v), 25 mg/L streptomycin, and 15 mg/L potassium penicillin G and supplemented with 1 mg/mL collagenase type 1 (Sigma) (pH 7.4; 4 mL/g AAT). Tubes were incubated at 37°C with gentle shaking for 40 min; thereafter, the fat suspension was filtered through a nylon cloth and centrifuged (30 sec at 400 rpm) at room temperature. Infranatants were aspirated and adipocytes washed with 10 mL of fresh sterile Krebs-Ringer-MOPS-BSA medium and centrifuged (3 times) as described above. Cells were then diluted with 3-4 mL of sterile Dulbecco's Modified Eagle's Medium (Sigma) (supplemented with 1% BSA, 1% FCS (v/v) and antibiotics (see above), pH 7.4 (incubation medium)) and counted. Cell samples were then diluted with incubation medium to yield ~200,000 adipocytes/900 mL distributed in 15 mL conical tubes and incubated for 45 min at 37°C, in a 95% O_2_-5% CO_2_ atmosphere without (basal) or with insulin (0.1–10 nM, Novo Nordisk Pharma AG, Switzerland) [29]. At the end of the incubation period, aliquots of media were carefully separated and kept frozen (−20°C) to measure LEP concentration. For this analysis, we used samples taken from 5 different experiments performed in 6 replicates.

### 2.5. AAT RNA Isolation and Real-Time Quantitative PCR

Total RNA was isolated from AAT pads of all experimental groups by a modification of the single-step, acid guanidinium isothiocyanate-phenol-chloroform extraction method (Trizol; Invitrogen, Life Tech., USA; cat. no. 15596-026) [32]. The yield and quality of extracted RNA were assessed by 260/280 nm optical density ratios and electrophoresis in denaturing conditions on 2% agarose gel. One microgram of total RNA was reverse-transcripted using random primers (250 ng) and Superscript III Rnase H-Reverse Transcriptase (200 U/*μ*L Invitrogen, Life Tech, USA; cat no. 18989-093). For quantitative real-time PCR, the following primers were applied: *β*-actin (ACTB) (R) 5′-ACCCTCATAGATGGGCACAG-3′, (F) 5′-AGCCATGTACGTAGCCATCC-3′ (115 bp) (GenBank accession number: NM_031144); LEP (R) 5′-CTCAGCATTCAGGGCTAAGG-3′, (F) 5′-GAGACCTCCTCCATCTGCTG-3′ (192 bp) (GenBank accession number: NM_013076); PAI-1 (R) 5′-TCTCCAGGGGCCCTCTGAGGT-3′, (F) 5′-TGCCCCTCTCCGCCATCACC-3′ (141 bp) (GBAN: NW_047370); IRS-1 (R) 5′-ACGGTTTCAGAGCAGAGGAA-3′, (F) 5′-TGTGCCAAGCAACAAGAAAG-3′ (176 bp) (GenBank accession number: NM_012969); IRS-2 (R) 5′-CCAGGGATGAAGCAGGACTA-3′, (F) 5′-CTACCCACTGAGCCCAAGAG-3′ (151 bp) (GenBank accession number: AF087674); glucose transporter (GLUT)-4 (R) 5′- TGGACGCTCTCTTTCCAACT-3′, (F) 5′-GCTTCTGTTGCCCTTCTGTC-3′ (166 bp) (GenBank accession number: NM_012751).

Two *μ*L of the reverse transcription mix were amplified by the QuantiTect Syber Green PCR kit (Qiagen Inc., CA, USA, cat. No. 204143, 0.5 mM of each specific primer and the Light Cycler Detection System, MJ Mini Opticon, Bio-Rad, CA, USA). PCR efficiency was ~1. Threshold cycles (Ct) were measured in separate tubes and in duplicate. The identity and purity of the amplified product were checked by electrophoresis on agarose minigels, and the melting curve was analysed at the end of amplification. Differences in cycle threshold (Ct) were calculated in every sample for each gene of interest as follows: Ct gene of interest and Ct reporter gene. ACTB, whose mRNA levels did not differ between control and test groups, was used as reporter gene. Relative changes in the expression level of one specific gene (ΔΔCt) were calculated as ΔCt of the test group minus ΔCt of the control group, then expressed as 2-ΔΔCt.

### 2.6. Statistical Analysis

Data were analysed by ANOVA (two factors: diet and treatment), followed by post hoc comparisons with Fisher's test [33]. The nonparametric Mann-Whitney test was applied to analyse data on adipose tissue mRNA concentration [33]. Results are expressed as mean ± SEM, and differences were considered significant when *P* values were below 0.05.

## 3. Results

### 3.1. Body Weight and Calorie Intake

All experimental groups had comparable body weights both at the beginning and after the 3-week study period; thus, they also showed a comparable increase in body weight (Table 1). Although animals receiving PIO have also increased significantly their body weight, their final values were significantly lower than those of PIO-untreated rats (Table 1).

Comparable amounts of daily energy intake were recorded over the 3-week experimental period in animals of all groups regardless of treatment or diet (Table 1).

### 3.2. Circulating Biomarker Levels 

#### 3.2.1. Metabolites and TBARS

 Rats fed with the FRD had significantly (*P* < 0.05 versus CD rats) higher plasma concentration of glucose, TG, NEFA, and TBARS (Table 2), thus indicating abnormal carbohydrate and lipid metabolism as well as an increased OS. PIO administration to FRD rats effectively prevented the development of all these abnormal changes (Table 2). 

#### 3.2.2. Hormone and Adipokine Levels

 Plasma levels of insulin and of all adipokines measured increased significantly (*P* < 0.05 versus CD rats) in the FRD rats (Table 2), thus suggesting an adipose tissue and *β*-cell dysfunction. Coadministration of PIO to these rats significantly (*P* < 0.05) decreased plasma levels of insulin, leptin, and PAI-1 (Table 2).

### 3.3. AAT Mass and Adipocyte Characteristics

AAT mass was slightly but significantly (*P* < 0.05) larger in FRD than in CD rats (Table 3), a difference that was not longer observed in PIO-treated rats, regardless of the diet. A significantly (*P* < 0.05) smaller number of adipocytes was obtained by collagenase digestion of AAT pads from FRD than from CD rats (Table 3); this difference was not found in rats treated with PIO, regardless of the diet (Table 3). However, the number of adipocytes obtained from AAT pads was significantly (*P* < 0.05) lower in FRD than in FRD-PIO group (Table 3). 

**Figure 1:**
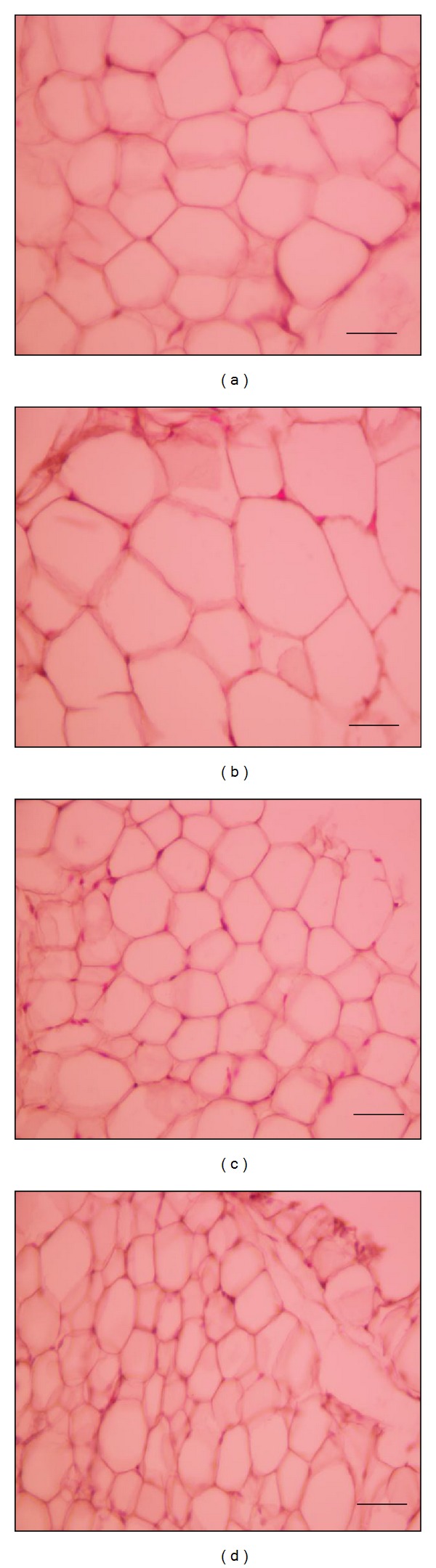
Representative fields of AAT from CD (a), FRD (b), CD-PIO (c), and FRD-PIO (d) rats stained with hematoxylin eosin (scale bar: 50 *μ*m; magnification: ×400).

These data closely correlate with changes observed in the size/volume of adipocytes (Figures 1(a)–1(d)). In fact, the AAT adipocytes of FRD rats were significantly (*P* < 0.05) larger (diameter and volume) than adipocytes of CD rats (Table 3). Additionally, adipocytes from PIO-treated rats were smaller than those of untreated rats regardless of the diet administered (Table 3). 

### 3.4. FRD-Induced Changes in AAT Leptin Content and Release: PIO Effect

The LEP content of AAT in FRD rats was significantly higher (*P* < 0.05) than in CD animals: 9.08 ± 2.06 versus 4.13 ± 0.23 ng/ng DNA (*n* = 7/8 rats per group). Although PIO treatment did not modify LEP content in CD rats (3.35 ± 0.96 ng/ng DNA; *n* = 7 rats), it fully prevented the increase induced by FRD (5.33 ± 1.12 ng/ng DNA; *P* < 0.05; *n* = 8 rats). 

**Figure 2:**
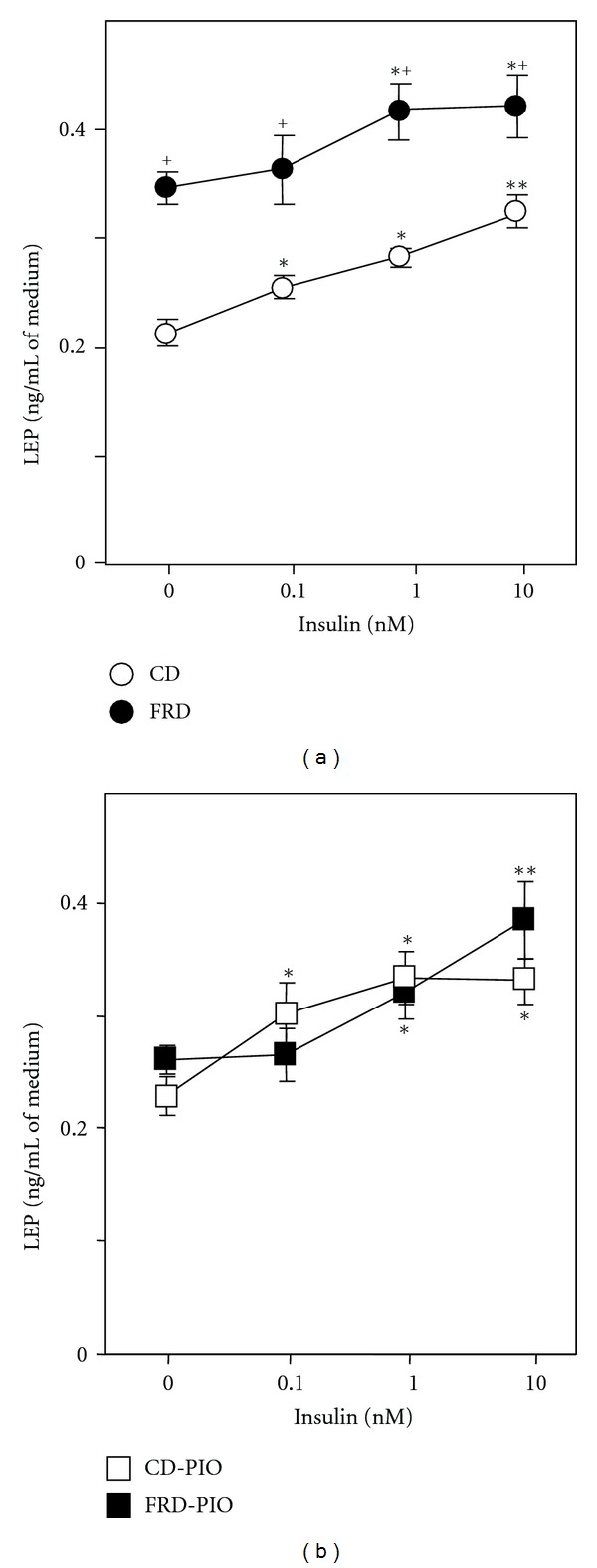
Effects of increasing concentrations of insulin on leptin release by adipocytes isolated from AAT of untreated CD and FRD rats (a) and from CD-PIO and FRD-PIO rats (b). Means ± SEM (*n* = 5 different experiments, with 6 replicates per condition). *P < 0.05 versus respective 0 nM insulin; **P < 0.05 versus 0.1 nM insulin; ^+^ *P* < 0.05 versus CD or CD-PIO in similar condition.

Adipocytes isolated from AAT of all experimental groups and incubated either without (baseline) or with increasing concentrations of insulin (0.1 to 10 nM; Figure 2) released leptin in a concentration-dependent fashion. This release was significantly higher in adipocytes isolated from FRD rats in any condition tested (Figure 2(a)). However, the threshold for insulin-induced leptin release in adipocytes from these rats shifted to the right (decreased insulin sensitivity), that is, a significant increase in leptin release (*P* < 0.05 versus baseline) started at an insulin concentration 10 times greater (1 versus 0.1 nM) in adipocytes from FRD rats than in those isolated from CD rats (Figure 2(b)). 

PIO coadministration did not affect leptin release in CD rats but it cut down its release in adipocytes from FRD rats to values comparable to those recorded in adipocytes from CD rats (Figure 2(b)), thus removing the difference between groups. However, PIO failed to fully correct impaired insulin sensitivity in FRD adipocytes (Figures 2(a) and 2(b)). 

### 3.5. Adipokines, IRS-1, and IRS-2 Gene Expression in AAT

**Figure 3:**
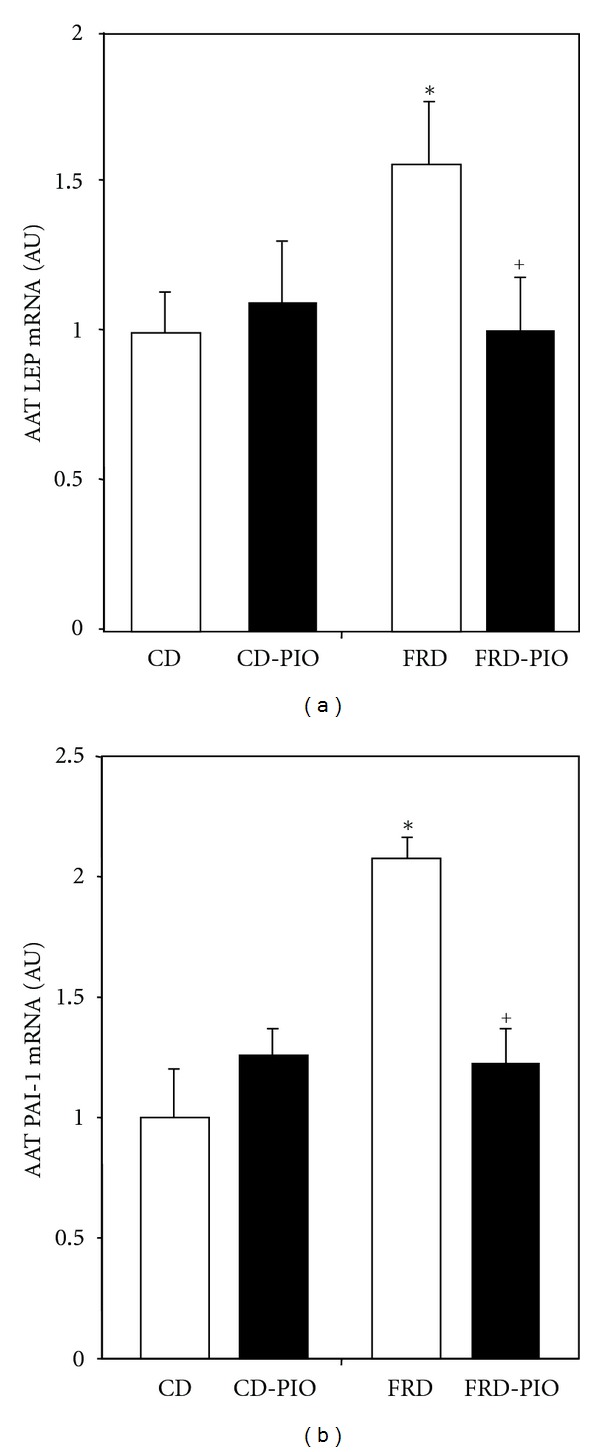
LEP (a) and PAI-1 (b) mRNA abundance in AAT pads from CD and FRD rats without or with PIO treatment. Data (expressed in arbitrary units: AU) were normalized to the levels of ACTB, and then presented as relative to values obtained in fat pads from CD rats. Means ± SEM (*n* = 5/6 pads per group). *P < 0.05 versus CD; ^+^ *P* < 0.05 versus FRD.

**Figure 4:**
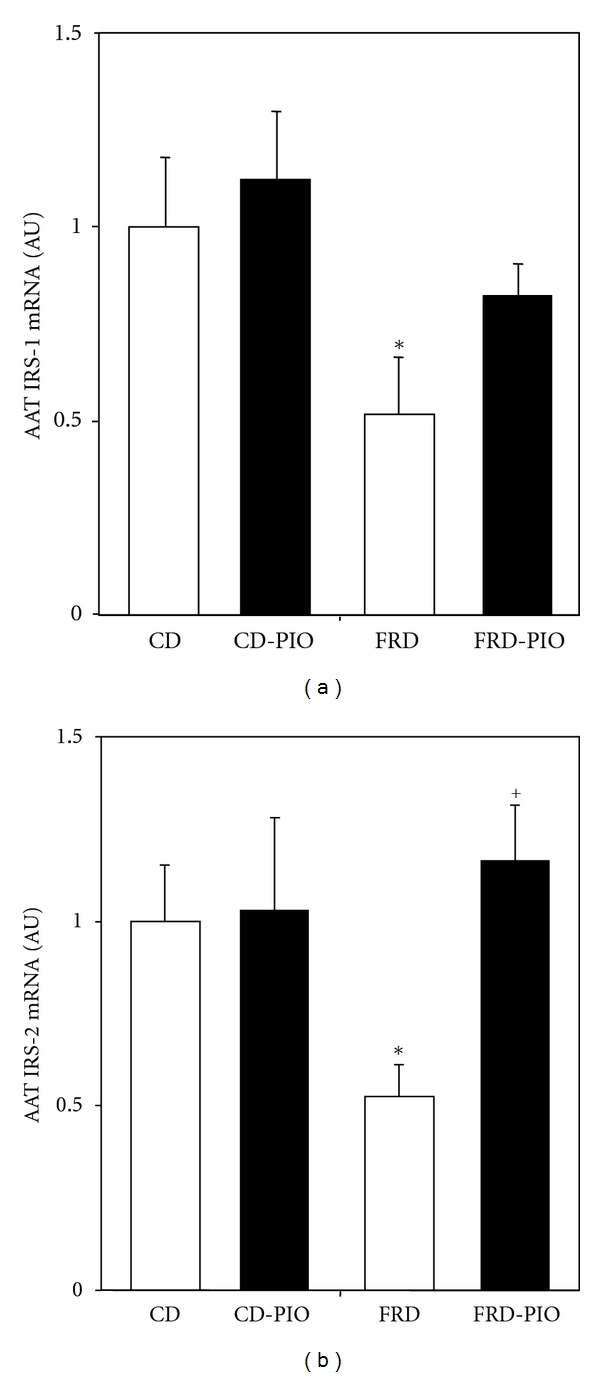
AAT IRS-1 (a) and IRS-2 (b) mRNA expression in pads from CD and FRD rats without or with PIO treatment. Data (expressed in arbitrary units: AU) were normalized to the levels of ACTB, and then presented as relative to values obtained in fat pads from CD rats. Means ± SEM (*n* = 5/6 pads per group). *P < 0.05 versus CD; ^+^ *P* < 0.05 versus FRD.

The mRNA content of LEP and PAI-1 in AAT was significantly (*P* < 0.05) higher in FRD than in CD rats (Figures 3(a) and 3(b), resp.). Although PIO administration to CD rats did not affect LEP and PAI-1 gene expression (versus CD rats), it abolished the enhanced expression of both genes in FRD rats (Figures 3(a) and 3(b), resp.). AAT IRS-1 and IRS-2 mRNA expression was significantly lower (*P* < 0.05) in FRD than in CD rats (Figure 4, both panels). Interestingly, PIO treatment partially and fully (*P* < 0.05) abolished the detrimental effect of FRD on AAT IRS-1 and IR-2 mRNA concentration, respectively (Figures 4(a) and 4(b)). Neither treatment- nor diet-dependent changes in AAT GLUT-4 mRNA expression were found (data not shown).

## 4. Discussion

Our current data support the reliability of the FRD rat model for its capacity to induce multiple metabolic and endocrine dysfunctions [14, 15]. Many of these changes are similar to those present in the human phenotype of MS [4–7, 34] which makes this animal model a useful tool to either study the production mechanism of such changes or to test the effectiveness of different treatment strategies. In fact, these animals portrait high plasma concentrations of metabolites and OS markers (glucose, NEFA, TG, and TBARS), insulin, and adipokines (LEP and PAI-1); these abnormally high levels of biomarkers support those reported in different studies [4–7, 14, 15, 35] and demonstrate the existence of several metabolic and tissue dysfunctions such as (a) impaired insulin sensitivity, (b) abnormal glucose and lipid metabolism, and (c) AAT dysfunction. Nonetheless, the present study selectively focused attention on the AAT dysfunction.

Since no significant differences were recorded in energy intake among the experimental groups, the lower final body weight of animals receiving PIO might be ascribed to a different utilization of metabolic substrates induced by this treatment. The changes described above in circulating metabolic and endocrine markers as well as those analysed below lend support to this assumption. 

AAT from FRD rats underwent significant changes in its mass (enlarged), adipocyte morphology (increased size), adipokine content (high leptin concentration), gene expression (enhanced LEP and PAI-1 mRNA abundance), and intracellular insulin mediators (decreased expression of IRS-1 and IRS-2 genes). Their adipocytes also displayed *in vitro *higher baseline and insulin-stimulated leptin release together with a decreased sensitivity to this stimulus. Altogether these changes indicate that FRD induces serious and multiple adipocyte dysfunctions whose development was effectively prevented by coadministration of PIO. 

The decrease in plasma insulin levels recorded in FRD rats treated with PIO indicates that the overall insulin sensitivity was significantly improved; however, the decreased sensitivity of the adipocytes from these animals to insulin stimulation was not fully corrected by this treatment. This latter effect could be mechanistically associated to the fact that PIOpartially corrected the impaired IRS-1 signalling in AAT; thus, these data suggest that AAT is not the main component of the overall insulin resistance in our rat model and that other mechanism/s rather than changes in insulin sensitivity could be involved in the FRD-induced AAT dysfunction. 

Because leptin is an active regulator of insulin activity, its high production (plasma levels and adipocyte release) could play a key role in the mechanism whereby FRD induces the alterations described above. In fact, high leptin levels affect insulin binding to its receptor [36] and reduce IRS-1/2 intracellular mediators downstream [37, 38], thereby potentiating and perpetuating overall insulin resistance. These data could explain the relationship between the high serum levels of leptin and reduced IRS-1/2 intracellular mediators, as well as the overall impaired insulin sensitivity present in our FRD rats. 

Regarding the potential role of PPAR-*γ* receptors in the production mechanisms of the abnormalities induced by FRD in AAT, PIO is an agonist of these receptors [26, 39–41] that exerts multiple effects on liver, muscle, and adipose tissue function [26, 27, 39, 40], including their sensitivity to insulin [27, 41]. In our case, its coadministration with the FRD effectively prevented the development of almost all the alterations induced by this diet. Thus, it is tempting to speculate that (a) PPAR-*γ* receptors could play a significant role in the mechanism whereby FRD induces AAT metabolic and endocrine dysfunctions (and probably in other tissues) and (b) such role could be exerted, at least partly, by improving insulin sensitivity.

As previously mentioned, however, we cannot discard that other mechanisms are probably involved in the PIO-preventive effect recorded in this study. In this regard, FRD intake induces metabolic and endocrine dysfunctions by enhancing OS [14, 42], as we previously reported [14] and currently confirmed. PPAR-*γ* agonists can decrease OS both *in vitro *[43, 44] and *in vivo *[45] and also increase the expression of catalase [46]. In our case, PIO coadministration significantly decreased the serum TBARS levels. Thus, perhaps the first event triggered by the FRD is an increase in OS rate that secondarily could impair insulin sensitivity, being these two effects responsible for the development of all the abnormal changes associated to its administration. Our current design, however, cannot completely define whether these are concurrent or sequential effects, one being primary in relation to the other. Otherwise, thiazolidinediones could also exert metabolic effects through PPAR-*γ*-independent mechanism [47–49], such as the effective antagonism on glucocorticoid receptor [50]. Consequently, PIO could display through this path an additional cooperative antidiabetic effect [50].

## 5. Conclusions

Our data firmly demonstrate that the multiple deleterious effects of FRD administration during 3 weeks upon the AAT function in normal rats can be largely counteracted by coadministration of pioglitazone. The PPAR-*γ* agonist preventive effect could be ascribed either to its action upon tissue insulin sensitivity and/or its antioxidant effect. The present data strongly suggest that these receptors play an active role in the mechanism whereby FRD exerts its deleterious effect upon metabolic and endocrine AAT functions. Nevertheless, the effectiveness of PIO treatment on FRD-elicited AAT dysfunction should not be solely ascribed to its PPAR-*γ* agonistic property. Otherwise, it also suggests that the development of new PPAR-*γ* agonists devoid of undesirable side effects of those currently available might be a useful tool to neutralize the damaging effect of excessive fructose consumption.

## Figures and Tables

**Table 1:** Initial and final (on third week after diet/treatment) rat body weight (BW) and 21-day average of energy intake.

	CD	CD-PIO	FRD	FRD-PIO
Initial BW (g)	192.44 ± 2.41	195.33 ± 6.37	189.78 ± 3.42	190.95 ± 3.18
Final BW (g)	301.83 ± 11.82	277.72 ± 9.31*	296.14 ± 6.44	270.78 ± 9.55^∗+^
Energy intake (cal/day/100 g BW)	25.91 ± 0.98	24.53 ± 2.56	28.82 ± 2.12	29.11 ± 2.83

Values are means ± SEM, *n* = 7/8 rats per group.

*P < 0.05 versus CD values; ^+^ *P* < 0.05 versus FRD values.

**Table 2:** Circulating levels of several markers of the adipoinsular axis function and TBARS.

	CD	CD-PIO	FRD	FRD-PIO
Glucose (mM)	7.16 ± 0.27	6.78 ± 0.25	8.27 ± 0.23*	7.11 ± 0.21^+^
Insulin (ng/mL)	0.75 ± 0.03	0.71 ± 0.08	1.13 ± 0.05*	0.89 ± 0.13^+^
Triglyceride (g/L)	1.11 ± 0.09	1.01 ± 0.06	1.74 ± 0.12*	1.19 ± 0.09^+^
NEFA (mM)	0.59 ± 0.04	0.56 ± 0.03	0.77 ± 0.05*	0.48 ± 0.05^+^
LEP (ng/mL)	4.77 ± 0.35	3.31 ± 0.25	6.67 ± 0.57*	4.88 ± 0.55^+^
PAI-1 (ng/mL)	1.59 ± 0.22	2.12 ± 0.37	3.72 ± 0.51*	2.01 ± 0.42^+^
TBARS (pmol/mg)	69.02 ± 5.09	79.15 ± 3.31	88.85 ± 3.13*	64.88 ± 9.89^+^

Values are means ± SEM, *n* = 7/8 rats per group.

*P < 0.05 versus CD values; ^+^ *P* < 0.05 versus FRD values.

**Table 3:** Abdominal adipose tissue (AAT) characteristics in male rats fed either a CD or FRD combined or not with PIO treatment.

	CD	CD-PIO	FRD	FRD-PIO
AAT mass (g)	1.87 ± 0.21	1.95 ± 0.28	2.48 ± 0.17*	2.11 ± 0.21
Cell number (×10^6^) per g AAT	2.94 ± 0.05	3.05 ± 0.29	2.08 ± 0.11*	2.59 ± 0.21^+^
AAT cell diameter (*μ*m)	50.11 ± 0.79	43.88 ± 1.01*	57.37 ± 0.69*	42.12 ± 1.78^∗+^
AAT cell volume (*μ*m^3^ × 10^3^)	66.28 ± 2.89	47.32 ± 3.88*	92.51 ± 3.22*	44.11 ± 5.21^∗+^

Values are means ± SEM, *n* = 7/8 rats per group.

*P < 0.05 versus CD values; ^+^ *P* < 0.05 versus FRD values.
